# A Review of Quartz Crystal Microbalance-Based Mercury Detection: Principles, Performance, and On-Site Applications

**DOI:** 10.3390/s25165118

**Published:** 2025-08-18

**Authors:** Kazutoshi Noda, Kohji Marumoto, Hidenobu Aizawa

**Affiliations:** 1 Environmental Management Research Institute (Previous Affiliation), National Institute of Advanced Industrial Science and Technology (AIST), Onogawa 16-1, Tsukuba 305-8569, Ibarak, Japan; 2 Research Organization of Science and Technology, Ritsumeikan University, 1-1-1 Nojihigashi, Kusatsu 525-8577, Shiga, Japan; 3 Environmental Chemistry Section, Department of Environment and Public Health, National Institute for Minamata Disease (NIMD), Hama 4058-18, Minamata 867-0008, Kumamoto, Japan; koji_marumoto@env.go.jp; 4 Environmental Biotechnology Research Group, Environmental Management Research Institute, National Institute of Advanced Industrial Science and Technology (AIST), Onogawa 16-1, Tsukuba 305-8569, Ibaraki, Japan; hide-aizawa@aist.go.jp

**Keywords:** mercury, sensor, artisanal and small-scale gold mining (ASGM), quartz crystal microbalance (QCM), environmental measurement

## Abstract

Mercury (Hg) is a globally recognized toxic element, and the Minamata Convention on Mercury entered into force in 2017 to address its associated risks. Under the United Nations Environment Programme, international efforts to reduce Hg emissions and monitor its environmental presence are ongoing. In support of these initiatives, we developed a simple and rapid mercury detection device based on a quartz crystal microbalance (QCM-Hg sensor), which utilizes the direct amalgamation reaction between Hg and a gold (Au) electrode. The experimental results demonstrated a proportional relationship between Hg concentration and the resulting oscillation frequency shift. Increased flow rates and prolonged measurement durations enhanced detection sensitivity. The system achieved a detection limit of approximately 1 µg/m^3^, comparable to that of commercially available analyzers. Furthermore, a measurement configuration integrating the reduction-vaporization method with the QCM-Hg sensor enabled the detection of mercury in aqueous samples. Based on the experimental results and the gas-phase detection sensitivity achieved to date, concentrations as low as approximately 0.05 µg/L appear to be detectable. These findings highlight the potential of the QCM-Hg system for on-site mercury monitoring. This review aims to provide a comprehensive yet concise overview of QCM-Hg sensor development and its potential as a next-generation tool for environmental and occupational mercury monitoring.

## 1. Introduction: Environmental Mercury Issues and Monitoring Needs

While the Industrial Revolution enriched human life, it also led to pollution problems caused by various chemical substances emitted from factories. In particular, heavy metals such as lead, arsenic, and mercury are known to negatively affect human health [1].

Mercury predominantly exists in its elemental form as a liquid metal under ambient temperature conditions and is naturally present within mineral matrices and soils. From these terrestrial reservoirs, mercury is gradually mobilized and released in trace amounts into fluvial and marine systems, primarily through subsurface hydrological pathways. Additionally, a portion of mercury volatilizes and is emitted into the atmosphere. Due to its ability to undergo long-range atmospheric transport and subsequent global dispersion, mercury is classified as a contaminant of global environmental concern. It has a high capacity to accumulate in the ecosystem and causes significant negative effects on both human health and the environment [2,3]. Minamata disease, first identified in the 1950s in the area surrounding Minamata Bay and Yatsushiro Sea, Japan, is one of the most internationally recognized pollution-related illnesses.

The Minamata Convention on Mercury was adopted at the Conference of Plenipotentiaries held in Kumamoto Prefecture in 2013. The treaty came into effect in 2017 [4,5,6]. Its main provisions are as follows:

1. Regulation of mercury supply sources and trade.
2. Regulation of products and manufacturing processes.
3. Control of emissions to the atmosphere.
4. Waste management.
5. Protection of human health and the environment.

This convention encourages countries to cooperate in reducing the use and emission of mercury to protect both the global environment and human health. In 2016, the Japanese government implemented the Act on Preventing Environmental Pollution of Mercury, which includes measures such as mercury reduction and waste management, and ratified the Minamata Convention on Mercury. The government is responsible for managing and monitoring mercury emissions and concentrations at waste treatment facilities [4].

**Figure 1:**
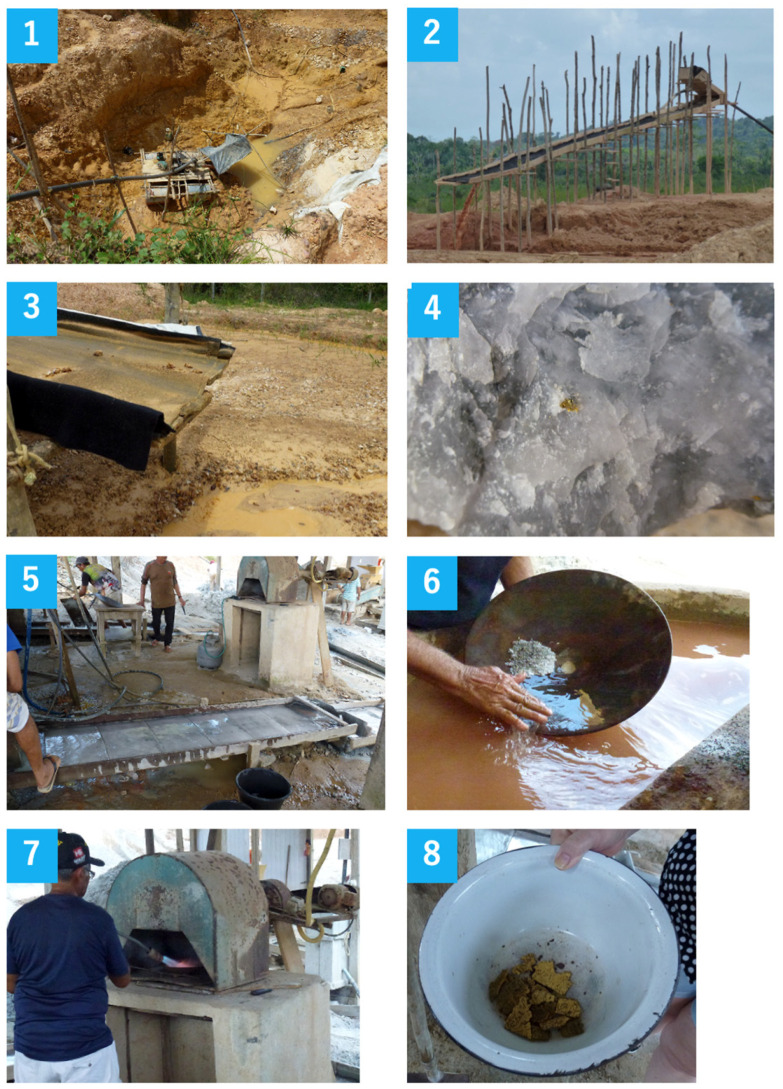
Representative scenes from artisanal and small-scale gold mining (ASGM) operations. (**1**) Mining of gold-bearing soil. (**2**–**3**) Washing and gravity separation of placer deposits using water channels and sluice boxes. (**4**) Gold-bearing quartz rock extracted for further processing. (**5**) Crushed rock is washed over a mercury-coated copper plate, enabling mercury to capture fine gold particles. (**6**) Scraping of the mercury–gold amalgam from the plate surface. (**7**) Heating of the amalgam using a burner to vaporize mercury. (**8**) Residual sponge gold obtained after vaporization.

One of the major sources of mercury released into the atmosphere is “artisanal and small-scale gold mining (ASGM)” (Figure 1).

ASGM refers to gold mining conducted by individual miners or small enterprises with limited capital investment and production capabilities (Minamata Convention Articles 2 and 7). Atmospheric mercury pollution is not limited to high-level local contamination around these mining areas. Currently, mercury emissions in Japan account for about 1% of the global total. According to the latest Global Mercury Assessment report by the United Nations Environment Programme (UNEP), mercury emissions from ASGM in Southeast Asia, Africa, and South America exceed 1000 tons per year, making up more than 40% of total anthropogenic atmospheric emissions [7]. Furthermore, releases into the pedosphere and hydrosphere are also estimated to exceed 1000 tons per year.

The large mercury emissions from ASGM are primarily due to the use of the gold amalgamation method for extracting gold. This method is extremely low-cost and can be easily used by anyone. It is mainly employed in mountain and forest regions of Southeast Asia and South America. In addition to environmental pollution, concerns have been raised about the health hazards to workers due to mercury exposure [8,9,10]. However, there is limited data on the health risks to workers in these areas because of the lack of detailed surveys. Local pollution is spreading, resulting in widespread, low-level contamination, primarily through the air and groundwater. The World Health Organization (WHO) European Committee recommends that mercury concentrations in the work environment should be less than 1 µg/m^3^ (about 0.12 ppb) [7].

When measuring mercury in the gas phase, detection of mercury compounds present at extremely low concentrations is typically carried out using mercury analyzers based on atomic absorption spectrometry (AAS) [11,12,13,14]. However, this method requires skilled operation and is not readily accessible for routine use by non-experts. Portable instruments based on AAS are also commercially available. However, due to their high cost and other limitations, they are not easily accessible, particularly in regions such as those engaged in ASGM.

On the other hand, the gas detection tube method [15,16], which relies on chemical reactions that produce a color change to detect mercury, is a simple and inexpensive measurement technique that anyone can use. For this reason, it is widely employed not only for mercury detection but also in various other fields. However, it lacks sufficient sensitivity for measuring mercury concentrations in the atmosphere. In particular, the World Health Organization’s environmental guideline value for mercury in the air 1 µg/m^3^ cannot be detected using the gas detection tube method.

The allowable concentration of mercury in aqueous phases varies by country. In Japan, the allowable concentration of mercury in drinking water is specified in the Water Quality Standards as “mercury and its compounds must not exceed 0.0005 mg/L (0.5 µg/L).” The wastewater standards stipulate that “mercury, alkyl mercury, and other mercury compounds must not exceed 0.005 mg/L (5 µg/L), provided that alkyl mercury compounds are not detected” using the reference method [17]. This means that the drinking water standard is 10 times stricter than the wastewater standard. Therefore, immediate measures to reduce mercury emissions are required. In rare cases, drinking water from groundwater sources may contain small amounts of mercury. This is often due to naturally higher mercury concentrations in certain areas, influenced by soil and other natural sources. As a result, there is a significant demand for mercury measurement in soil and water quality within Japan, and a method that can be easily performed on-site is desired.

When measuring mercury in the liquid phase, the reduction-vaporization method [18,19] is primarily employed. Typically, the mercury present in water is not in its metallic form but as mercury compounds. Therefore, a ferrous tin chloride (SnCl_2_) solution is added to the sample water (Hg^2+^) to reduce it to zero-valent metallic mercury. The solution is then bubbled to purge the metallic mercury. The purged metallic mercury is then analyzed to determine the mercury concentration using AAS. The gas detector tube method can also be used to measure the vaporized metallic mercury in this process [20]. The sensitivity of AAS is high, allowing for the detection of extremely small amounts of metallic mercury, making it suitable for measuring water quality standards for tap water in Japan. The gas detector tube method, on the other hand, is capable of measuring the Japanese effluent standard values.

To reduce environmental risks from mercury, a simple and rapid detection system for mercury in the environment is essential. In particular, there is a demand for a highly sensitive system that can be easily used by anyone at a low cost. To address this need, we have developed a highly sensitive method capable of detecting low concentrations of mercury using quartz crystal microbalance (QCM) [21,22]. This new detection method offers sensitivity at the ng level, enabling the detection of low mercury concentrations. Furthermore, this method can detect not only mercury in the atmosphere but also mercury in aqueous solutions [23]. While the measurement cost is comparable to that of the gas detection tube method, its detection sensitivity is more than 10 times greater. We have been improving this method to make it practical as a sensor that can be easily used by anyone, anytime, and anywhere.

This review aims to provide a comprehensive overview of the QCM-based mercury detection system developed with a focus on the authors’ recent contributions, highlighting its measurement principle, experimental configurations, detection performance, and application potential. While this review includes recent experimental findings, its primary goal is to contextualize the QCM-Hg system among existing mercury detection approaches and explore its advantages for on-site environmental and occupational monitoring. This review is structured to first outline the working principle of QCM-based mercury detection, followed by its experimental implementation and performance evaluation.

In this study, various experiments were conducted to clarify the characteristics of the mercury detection method using QCM. The results highlight the unique features of this mercury detection approach utilizing QCM.

## 2. Principles of Quartz Crystal Microbalance for Mercury Detection

The principle of gas detection using QCM relies on converting changes in the mass adsorbed on the electrode surface of the quartz crystal into changes in frequency [24,25]. This principle is widely employed in gas and odor sensors. Generally, quartz crystal is extensively used as reference oscillators in electronic devices such as clocks, radios, and computers. The concept of utilizing quartz crystals as gas detection devices was pioneered by Sauerbrey. He discovered that when a substance adsorbs onto the quartz crystal electrode surface, the resulting change in mass leads to a shift in its frequency. This detection method is commonly referred to as the QCM method.

QCM is a sensor device capable of measuring ng-level mass changes on a quartz crystal wafer surface, expressed as a frequency shift. This frequency shift can be described using Sauerbrey’s equation as follows [26]:

Δ*f* = −2Δ*m*
*n*
*f*_0_^2^/(*A*
*µ*_*q*_^1/2^
*ρ*_*q*_^1/2^) (1)

where *f*_0_ is the resonant frequency, Δ*f* is the frequency shift, Δ*m* is the mass change in the QCM caused by adsorption or desorption on the electrode, *n* is the harmonic number, *A* is the area of the electrode coated on the crystal, *µ_q_* is the elastic coefficient (2.947 × 10^13^ gm^−1^ s^−2^), and *ρ_q_* is the crystal density (2.648 g cm^−3^).

When using QCM as a gas sensor, it is typically necessary to apply a detection film to the electrode surface of the quartz crystal resonator that readily adsorbs the target gas. This method leverages the characteristic behavior where the target gas adsorbs onto the detection film and subsequently desorbs from it. Therefore, it is crucial to develop a detection film that selectively adsorbs the target gas [27,28,29,30,31,32,33,34,35,36,37,38,39,40]. However, achieving selective detection is often challenging because substances other than the target gas can also adsorb onto the detection film. Moisture, in particular, frequently interferes with measurements, necessitating the implementation of measures to mitigate its impact.

In the mercury detection mechanism of QCM, the gold electrode of the QCM reacts directly with mercury in the air. This reaction is known as the amalgam reaction or amalgamation.

Here, we briefly explain the process by which mercury and gold form an amalgam alloy [41,42,43,44]. When metallic gold comes into contact with mercury, mercury atoms diffuse into the gold surface. This leads to the gradual dissolution of gold atoms into the mercury, resulting in the formation of a gold–mercury alloy known as an amalgam. The process typically occurs at room temperature and does not require any external energy input. Initially, a thin amalgam layer forms on the gold surface. As contact continues, more gold dissolves and the amalgam layer thickens. This phenomenon is widely utilized in gold extraction, particularly through the amalgamation method.

Due to this mechanism, QCM gold electrodes can generally be used for mercury detection without the need for additional surface modifications. Because of this, the system eliminates the need for film formation, making it a cost-effective solution. Moreover, the absence of a detection film minimizes interference from other gases, and the device is particularly resistant to the effects of moisture.

We developed a technique for the direct reaction between mercury and the gold electrode of the QCM through the phenomenon of “mercury–gold” amalgamation. Despite its simplicity, this system enables highly sensitive detection. We refer to the detection method based on this principle as the QCM-Hg sensor [45].

## 3. Experimental Implementation of QCM-Hg Sensors

Based on the previously described detection principle, a series of experiments was conducted to evaluate the performance and practical applicability of the QCM-based mercury sensor system. The following sections detail the experimental setup, sensor response characteristics, and the influence of environmental factors, with an emphasis on usability in real-world monitoring scenarios.

### 3.1. Detection of Mercury Vapor Using QCM Sensor

This section outlines the materials and experimental procedures used for mercury detection in the gas phase. The experiments were conducted to evaluate the performance and sensitivity of the QCM-Hg sensor under controlled conditions. Key aspects of the experimental setup, including the preparation of the QCM sensor, the generation of mercury-containing gas samples, and the measurement protocols, are described in detail.

**Figure 2:**
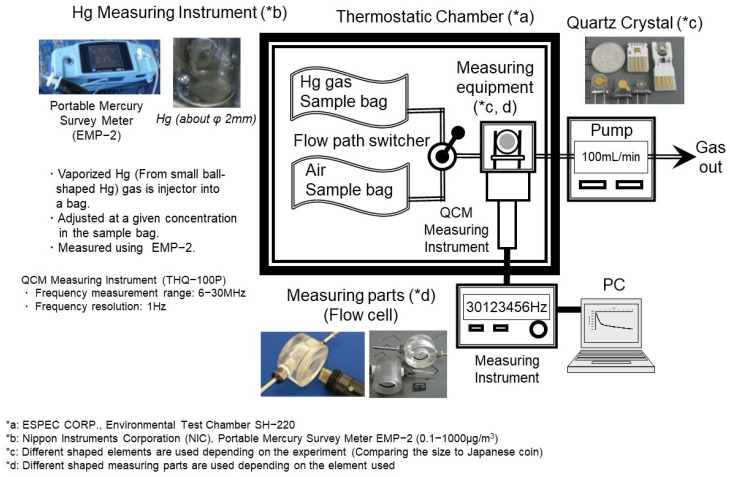
Schematic diagram of the experimental setup used for QCM-based mercury (Hg) gas detection. Hg gas is generated from a small metallic Hg source and introduced into a sample bag at a controlled concentration, with monitoring performed using a portable mercury survey meter (EMP-2, NIC) (*b). The sample bag is connected to a flow path switching system that allows alternation between Hg gas and air. Both gases are passed through a thermostatic chamber (SH-220, ESPEC) (*a), which houses the QCM sensor unit. The sensor contains a quartz crystal resonator (*c) placed in a flow cell (*d), and frequency shifts due to Hg adsorption are recorded using a QCM frequency measuring instrument (THQ-100P, SEIKO EG&G) and logged on a PC. Reprinted with permission from Ref. [21]. Copyright (2020) Publisher MYU K.K.

We conducted tests to determine the basic detection mechanism of the QCM-Hg sensor. Figure 2 presents the configuration of a representative experimental setup employed in the present study. In each experimental run, a new gold electrode for the quartz crystal was utilized. To ensure an efficient reaction between mercury in the air and the gold electrode of the QCM, the detection component was capped with a flow cell. Due to the compact size of the detection device, this configuration allowed for precise control over the reaction conditions. To maintain a constant temperature during each experimental run, most components of the experimental apparatus, including the quartz oscillator but excluding the frequency counter, PC, and suction pump, were placed in a thermostatic chamber (SH-220, ESPEC CORP., Osaka, Japan.), as shown in Figure 2. The gaseous mercury (Hg gas) and the QCM surface were both maintained at the same temperature. The Hg gas used in the test was generated by injecting a trace amount of mercury into a vaporization bottle and vaporizing it with a constant flow of carrier gas. The prepared Hg gas was injected into a sample bag, diluted with the reference gas, and then used as the sample gas for testing. Artificial air (N_2_ + O_2_) and nitrogen (reference air) were used as reference gases, with no significant difference observed in results between the two. The mercury concentration in the sample gas was measured using a mercury survey meter (EMP-2, Nippon Instruments Corporation, Kyoto, Japan.). This ensured accurate determination of the Hg gas concentration throughout the experiments.

It is worth noting that, in general, QCM-based gas sensors show increased frequency shifts as the measurement time and gas flow rate increase [46,47,48,49]. Based on this principle, it is expected that increasing the measurement time and flow rate may enable the detection of low concentrations of mercury vapor. The objective of this study is to develop a high-sensitivity, on-site mercury detector suitable for use under field conditions. Accordingly, measurement time and flow rate were determined with practical field applications in mind.

For measurement time, a duration of approximately 10 min was adopted, based on the typical measurement times (about 5 to 10 min) of gas detector tubes commonly used in field settings.

Regarding flow rate, a baseline of 100 mL/min was selected, considering the capabilities of commercially available, battery-operated, portable suction pumps used for field sampling. Given these conditions, the required sample bag volume was set to 1000 mL or larger.

Sample gas was prepared using bags such as Tedlar bags, which have low adsorption characteristics for mercury vapor. Additionally, experiments were conducted under varied measurement parameters to compare detection characteristics.

Based on these experimental results, this study aims to determine the optimal operating conditions that allow for reliable detection of mercury concentrations at or below environmental regulatory levels under actual field conditions. These mercury–gold reactions are quantified through changes in the oscillation frequency of the QCM and recorded on a computer.

### 3.2. Detection of Mercury in Aqueous Samples via Reduction Vaporization and QCM

This section describes the experimental procedures used for mercury detection in the liquid phase. The experiments aimed to evaluate the performance of the QCM-Hg sensor when detecting mercury dissolved in liquid samples. In the proposed detection method based on reduction vaporization, the viscosity of water does not influence the detection mechanism.

**Figure 3:**
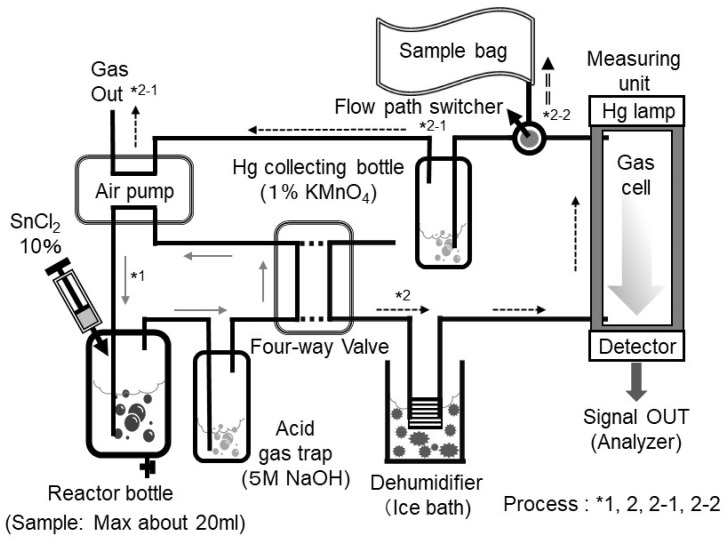
Structure of the Reduction Vaporization–Cold Atomic Absorption Spectrophotometry (Circulating–Open Aeration System). Reprinted with permission from Ref. [23]. Copyright (2021) Institute of Electrical Engineers of Japan.

The experimental setup using the reduction-vaporization method is shown in Figure 3. In this experiment, an instrument for mercury determination by reduction-vaporization atomic absorption spectrometry (circulation-open-air method, HG-201, manufactured by SANSO SEISAKUSHO Co, Tokyo, Japan) was utilized [50]. The QCM-Hg sensor leveraged the vaporization process of this instrument to conduct the experiment. Here, a widely used HgCl_2_ was employed as a simulated sample water. This HgCl_2_ solution was prepared using 0.1 M HNO_3_.

First, the solution was placed in a reaction bottle (containing Hg^2+^ in solution). Next, an appropriate amount of 10% SnCl_2_ solution (prepared with a 2 M HCl solution) was added to the reaction bottle. In this process, mercury in the solution is reduced to form metallic mercury by SnCl_2_ as follows:

HgCl_2_ + SnCl_2_ → Hg + SnCl_4_ (2)

Acid gases and other by-products generated during this reaction, which are not required for measurement, are trapped by passing the gases through a 5 M NaOH (referred to as the acid gas trap). This trap prevents interference with the QCM-Hg sensor measurement. The components of the air pump, reaction bottle, and acid gas trap are sealed using a four-way stopcock. In this setup, the air pump circulates the air at a flow rate of 1–1.5 L/min for 30 s (Refer to *1 in Figure 3). The purpose of this circulation is to homogenize the generated mercury gas. After homogenization, the four-way stopcock is switched, allowing the homogenized mercury gas to pass through a dehumidifier and enter the gas cell (measuring unit) (refer to *2 in Figure 3). Light irradiation from the light-emitting device (Hg lamp) causes absorbance to change according to the concentration of mercury gas within the light-absorbing cell. The change in absorbance is measured by a photodetector and converted into mercury concentration using a calibration curve. Following measurement, the mercury gas in the light absorption cell is absorbed by passing through a mercury trap bottle (containing 1% KMnO_4_ solution), ensuring the gas is removed and the atmosphere remains mercury-free (refer to *2–1 in Figure 3).

The experiment with the QCM-Hg sensor follows the same process up to the point before switching the four-way stopcock. The key difference occurs after switching the four-way stopcock. After switching, mercury gas is trapped in a sample bag (with a trap volume of approximately 1 L) before passing into the Hg collecting bottle (refer to *2-2 in Figure 3). The mercury gas in the sample bag remains in a vaporized state, meaning it can be detected using a measurement similar to that of the QCM-Hg sensor in the gas phase. It should be noted that this gas cell is not required when measurements are conducted using the QCM-Hg sensor. In this experiment, indoor air was used as the reference gas. For this purpose, a gold adsorption trap tube (manufactured by Nippon Instruments Co., Tokyo, Japan) was employed to remove the extremely small amount of mercury present in the air. The gold adsorption trap tube is capable of adsorbing trace amounts of mercury from the air (typically around several ng/m^3^) and is recognized as an official method in environmental monitoring [51]. The gas passing through this trap tube served as reference air. In this QCM-Hg sensor experiment, reference air was first flowed through to ensure no frequency shift (confirmation of a stable baseline). The sample bag was then switched to one containing mercury gas, and the frequency shift relative to the mercury concentration was measured. The aim of this experiment was to determine whether the QCM-Hg sensor could measure mercury concentrations at water quality reference levels (0.5 µg/L). A HgCl_2_ solution prepared at a concentration of 10 ng/mL was used. A 0.5 mL aliquot of this solution, containing 5 ng of mercury, was collected in a reaction bottle, to which approximately 10 mL of pure water was added as sample water. The mercury concentration in this sample water corresponds to the Japanese water quality standard concentration of 0.0005 mg/L (0.5 µg/L). Next, approximately 1 mL of a 10% SnCl_2_ solution was added to the reaction to facilitate reduction and vaporization. Assuming complete vaporization of the mercury, the mercury gas was collected in a sample bag (1 L), which corresponds to a gaseous mercury concentration of approximately 5 µg/m^3^.

**Figure 4:**
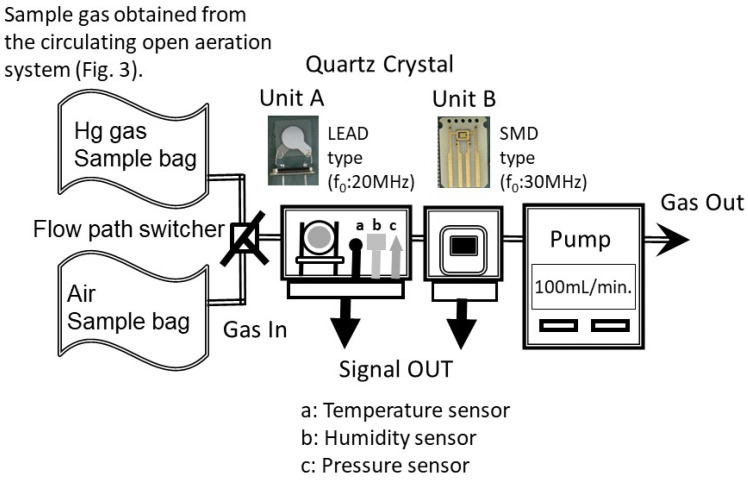
Basic experimental setup for the QCM-Hg sensor for liquid-phase measurement.

In this experiment, two different quartz crystals were used, and the measurement results were compared. One was a LEAD type quartz crystal (*f*_0_ = 20 MHz) and the other was a surface mount device (SMD) type quartz crystal (*f*_0_ = 30 MHz) (Figure 4). For each QCM-Hg sensor, mercury gas was aspirated at a flow rate of 100 mL/min. The measurement time was approximately 8–10 min, with a gas volume of around 0.8–1 L collected in the sample bag. Experiments were conducted under these conditions, and the frequency shift of each QCM-Hg sensor was measured. The experiment was performed five times, with a new crystal unit used for each measurement.

## 4. Performance Evaluation and Practical Considerations

This section summarizes the key performance metrics of the QCM-based mercury sensor, including its detection sensitivity, response time, and selectivity. In addition, we discuss practical considerations for on-site usage, such as long-term stability, reproducibility, and environmental robustness, with reference to the experimental results. These insights are essential for assessing the applicability of this technology in real-world monitoring contexts.

### 4.1. Influence of Sample Flow Rate on QCM Response

**Figure 5:**
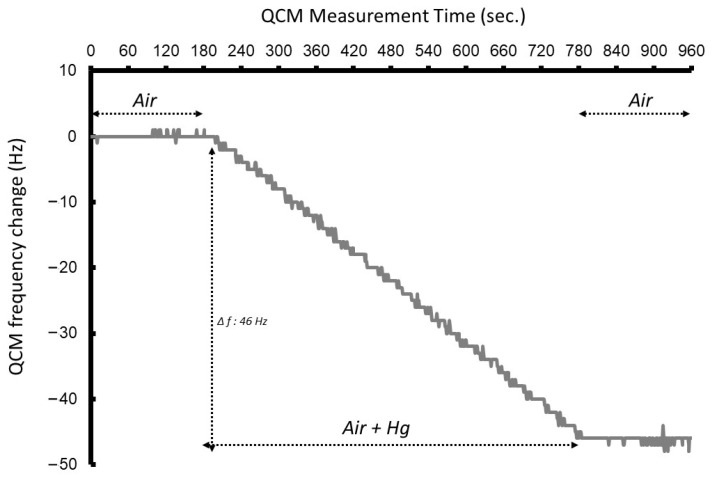
Frequency response of the QCM sensor to mercury vapor exposure in a test run. The detection mechanism is based on the decrease in the fundamental oscillation frequency of the quartz crystal due to the formation of a mercury–gold amalgam on the electrode surface (Δ*f* < 0). In this experiment, a frequency shift of 46 Hz was observed over 10 min of exposure to mercury vapor. Measurement conditions: QCM fundamental frequency = 30 MHz (surface-mounted device type); Hg concentration = 9.9 µg/m^3^; flow rate = 100 mL/min. Reprinted with permission from Ref. [21]. Copyright (2020) Publisher MYU K.K.

We conducted two experiments: one aimed at determining the detectable concentration range of mercury, and the other at investigating the response characteristics of the proposed detection method. In the response characteristics test, reference air was introduced at a constant flow rate into a flow cell containing the quartz crystal, and the resulting changes in oscillation frequency were monitored. When reference air (mercury-free) was supplied to the QCM, no reaction occurred, and thus, no frequency shift was observed. Subsequently, the reference air was replaced with a sample gas containing a defined concentration of mercury. The oscillation frequency of the QCM decreased as the mercury concentration increased. This behavior is attributed to the irreversible reaction between mercury and the gold electrode. Upon termination of the Hg gas supply and its replacement with reference air, the QCM frequency stabilized and ceased to decrease further. Initially, the response characteristics of the QCM to mercury were evaluated (Figure 5). In this test, gas was supplied at a flow rate of 100 mL/min. Each measurement was conducted for 10 min under these conditions, during which the oscillation frequency shift was recorded. Consequently, a total of 1000 mL of sample gas was delivered to the electrode surface of the QCM. The test results showed that the frequency decreased linearly over time at a constant rate. Subsequently, the maximum frequency shift (Δ*f*) was measured at various mercury concentrations. The results indicated a proportional relationship between the frequency shift and mercury concentration.

**Figure 6:**
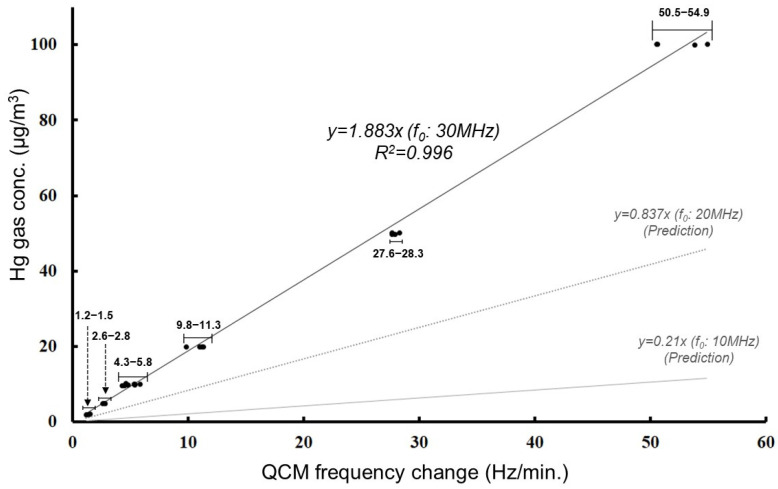
Calibration curve showing the relationship between QCM frequency change rate and mercury vapor concentration. A linear relationship was observed between the rate of frequency change (Hz/min) and the mercury concentration (µg/m^3^) for a quartz crystal sensor with a fundamental frequency of 30 MHz (SMD type). In the representative case shown in Figure 5, a 46 Hz frequency shift over 10 min corresponds to a rate of 4.6 Hz/min for a concentration of approximately 10 µg/m^3^. Using this trend, the expected sensitivity at 1 µg/m^3^ is approximately 0.46 Hz/min. Predicted calibration curves for quartz crystals with fundamental frequencies of 20 MHz and 10 MHz were also plotted based on Equation (1), demonstrating lower sensitivity with decreasing frequency. Measurement conditions: flow rate = 100 mL/min; Hg gas exposure = 10 min. Specifically, the measured ranges of gas concentrations were as follows (listed in ascending order of concentration): 1.9–2.1 µg/m^3^, 4.9–5.0 µg/m^3^, 9.7–10.2 µg/m^3^, 19.9–20.0 µg/m^3^, 49.8–50.2 µg/m^3^, and 100.0–100.3 µg/m^3^. Reprinted with permission from Ref. [21]. Copyright (2020) Publisher MYU K.K.

Based on these results, a calibration curve was constructed using the frequency shift data (Figure 6). The following is an approximate expression of the calibration curve under the test conditions.

y = 1.883 x(3)

y: Hg concentration (µg/m^3^)
x: frequency change (Hz/min)
(This equation is valid only within the scope of the experimental conditions.)

Under these test conditions, the frequency change is proportional to the Hg concentration. This indicates that, based on the calibration curve, the frequency change can be converted into Hg concentration. According to the calibration curve obtained under these experimental conditions, a concentration of 1 µg/m^3^ corresponds to an average frequency change of about 0.5 Hz/min. The detection limit presented here was calculated based on the frequency change (Δ*f*) obtained from the experiments and the measured gas concentration (µg/m^3^). In other words, since the frequency shift over a 10-min measurement is approximately 5 Hz, the detection of mercury at the WHO guideline concentration is considered to be straightforward.

**Figure 7:**
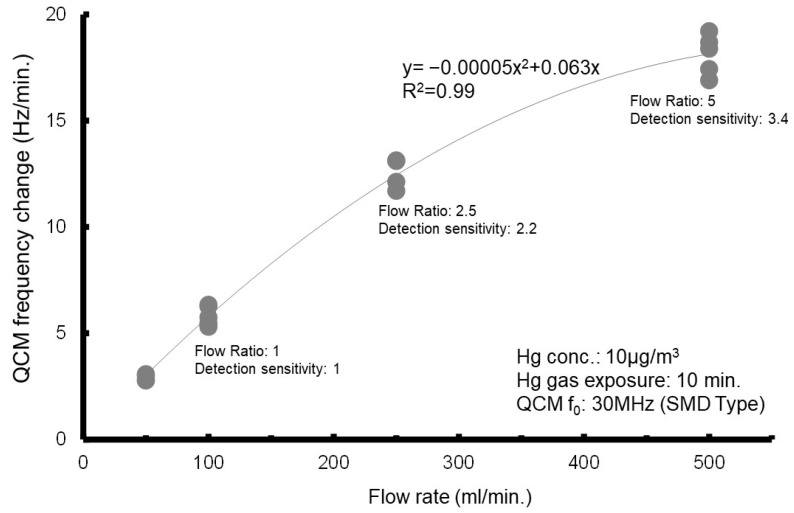
Relationship between quantities of change in frequency and flow rate. Reprinted with permission from Ref. [21]. Copyright (2020) Publisher MYU K.K.

Second, we conducted a test to investigate the relationship between the frequency change and the flow rate (Figure 7). The test results showed that the frequency change increases with an increasing flow rate. However, when the flow rate exceeds 250 mL/min, the rate of change becomes smaller. In this context, the flow velocity at the outlet of the gas cell was analyzed. At a flow rate of 100 mL/min, the flow velocity at the exit of the pipe (inner diameter: 2 mm) is approximately 0.5 m/s, increasing to about 1.3 m/s at 250 mL/min and about 2.6 m/s at 500 mL/min. The test results suggest that in the amalgamation reaction between mercury and the gold electrode, the amount of unreacted mercury increases, and the electrode Δ*f* relative to the flow rate decreases when the flow velocity of the Hg gas exceeds the reaction velocity of the mercury amalgamation process.

Third, we conducted a test to investigate the relationship between the frequency change and the measurement time. The test results showed that the frequency change increases with longer measurement times. The detection sensitivity also improves with an increasing fundamental oscillation frequency ([26], G. Sauerbrey’s equation). However, similar to the flow rate, there may be a critical point beyond which detection efficiency no longer improves significantly. The relationship between the quartz crystal electrode and the inlet is crucial in the current QCM measurement system, and the approximation equation derived from the experiments is mainly influenced by the flow rate. Based on these results, it is possible to utilize the approximation equation to convert frequency shifts into corresponding changes in mercury concentration, thereby enabling the creation of a conversion table. Commercially available gas detection tubes are typically accompanied by pre-prepared conversion charts that relate the length of color change in the reagent to gas concentration, and these are commonly used in field measurements. Similarly, in mercury detection using QCM, it is considered feasible to adopt a comparable approach for practical field applications.

This detection system utilizes the amalgam reaction between mercury and the gold electrode and exhibits high selectivity toward the target gas. This indicates that, due to the absence of an organic detection film, gases other than gaseous mercury are less easily adsorbed onto the gold electrode surface. Our previous test results show that an unmodified electrode (without a detection film formed) is less affected by substances other than water, trimethylamine, and sulfur-based substances [52]. Even when volatile organic compounds (VOCs) are present, the mercury–gold electrode reaction remains irreversible at room temperature, indicating that the amalgam reaction is less affected by VOCs. Therefore, the effects of substances other than mercury can be mitigated by continuously supplying only air or a non-mercury-containing gas to the QCM. However, measurements carried out over extended periods may lead to the adsorption of substances other than mercury, which could become immobilized on the surface of the quartz crystal. Measurements conducted for less than 1 h indicate that immobilization is less likely to occur. However, further system development is required to enable personal exposure monitoring for mercury over extended periods (approximately 8 h). To achieve this, further investigation into the effects of immobilization and measures to eliminate these effects will be necessary. The amalgam reaction may be particularly influenced by Suspended Particulate Matter (SPM) such as PM2.5. Since a disposable membrane filter (e.g., ADVANTEC’s DISMIC-25AS with a pore size of 0.2 µm) is used in EMP-2 (Nippon Instruments Corporation), a commercially available mercury survey meter, it is less affected by SPM. This suggests that such filters may help reduce these effects.

In general, QCM measurement systems are influenced by temperature and humidity, as indicated by changes in frequency (Δ*f*). The QCM-Hg system is no exception, exhibiting similar susceptibility to these environmental factors as other QCM-based measurement systems. This QCM-Hg is designed for measurements lasting approximately 10–15 min per sample. In a typical measurement environment, rapid fluctuations in temperature and/or humidity are unlikely to occur within such a short timeframe, so this QCM-Hg does not account for their influence. However, if the QCM-Hg system is significantly affected by variations in temperature or humidity, it is necessary to apply appropriate corrections to the frequency shift (Δ*f*) as needed. In such cases, the corrections (Δ*f*) can be performed by measuring the frequency change again under the same temperature and/or humidity conditions as those used for the reference air measurement.

Based on the experimental results obtained thus far, it has been shown that the system utilizing an SMD-type QCM (30 MHz) is capable of reliably detecting mercury concentrations below 1 µg/m^3^ (approximately 0.12 ppb), which is the level specified by the WHO guidelines for air quality. Mercury vapor at this concentration is not detectable using currently available gas detection tubes.

### 4.2. Comparative Evaluation of QCM Sensor Types

**Figure 8:**
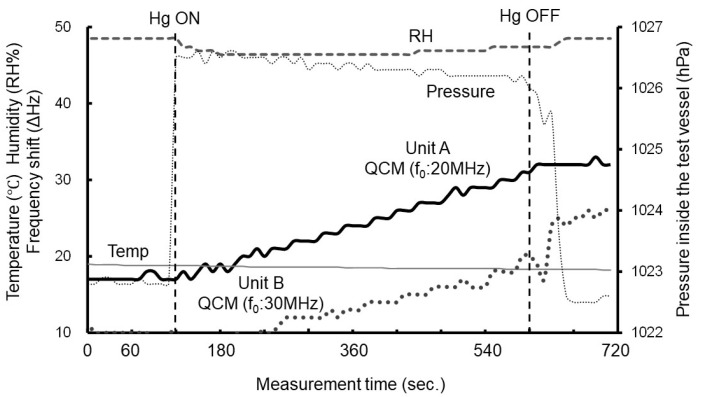
Example of frequency shift vs. measurement time (liquid phase). Reprinted with permission from Ref. [23]. Copyright (2021) Institute of Electrical Engineers of Japan.

An example of the experimental results obtained using the representative experimental setup employed in this study (Figure 4) is shown in Figure 8.

Similar to the previous results, we observed no change in frequency when air without mercury was used. When metallic mercury gas was passed through the quartz crystal, it was adsorbed onto the electrode surface, causing a decrease in the oscillation frequency (the graph shows the increase in frequency as the amount of change). Afterward, when air without mercury was introduced again, the change in the oscillation frequency ceased, and the frequency remained constant. From these results, we determined that the change in oscillation frequency observed in this experiment was caused by the vaporized metallic mercury (in its gaseous state) coming into contact with the gold electrode of the quartz crystal and forming an amalgam alloy, which resulted in a frequency shift. We confirmed that by employing this reduction-vaporization method with the QCM-Hg sensor, it is also possible to measure Hg^2+^ present in water. Additionally, we found that the mercury concentration measured in this experiment corresponds to the water quality standard concentration in the Japanese environment (0.5 μg/L), making it applicable for determining environmental standard levels.

The frequency variation within this range of mercury concentrations is discussed below. The experimental results under the current experimental conditions show that the frequency shift of the LEAD type (*f*_0_ = 20 MHz, Unit A) was 11 to 15 Hz (average 13 Hz, equivalent to 9 min of measurement time). For the SMD type (*f*_0_ = 30 MHz, Unit B), the frequency shift change was 7 to 12 Hz (average 9 Hz, equivalent to 9 min of measurement time). The amount of sample used in each experiment varied slightly, which is believed to contribute to the observed variation in frequency shifts during the measurements (the difference in sample amount also resulted in varying measurement durations).

As mentioned earlier (Section 3.2: Experiments on Mercury Detection in the Liquid Phase), the Hg concentration used in this experiment corresponds to approximately 5 µg/m^3^ when converted to a gas-phase concentration. For the SMD type in gas-phase experiments, the frequency shift over a 10-min period is estimated to be around 25 Hz (assuming 5 Hz per 1 µg/m^3^). In contrast, the LEAD type showed an average frequency shift of 13 Hz over a 9-min measurement, which is consistent with the results shown in Figure 6. In the case of the SMD type, the onset of the frequency change occurred approximately 2 min later than with the LEAD type. When using the onset of the frequency shift as the reference point, the effective measurement duration is estimated to be 6–8 min (average of 7 min), during which the observed frequency changes occurred.

Theoretically, the SMD type is more sensitive to detection than the LEAD type, but the actual measurement results (frequency change) showed that the SMD type exhibited approximately 0.7 times lower sensitivity (20 MHz: average 13 Hz, 30 MHz: average 9 Hz). The delay observed with the SMD type is attributed to the volume of the tube connecting the measurement equipment (approximately 100 mL). Additionally, the concentration of mercury reaching the SMD type may have been lower due to slight adhesion of mercury to containers, equipment, etc., along the measurement path. The reproducibility of the current study is consistent with previous experimental results in the gas phase. The LEAD type and SMD type used in this experiment have different structures. The LEAD type has electrodes on both sides that are more easily exposed to mercury gas. In contrast, the SMD type has electrodes on one side that are easily exposed to mercury gas, while the other side is less exposed to mercury gas.

In utilizing the QCM-Hg sensor, it is considered possible to calculate the mercury concentration even for shorter measurement times than those used in the experiment, by employing an algorithm that relates the mercury concentration to the slope of the oscillation frequency shift. The quartz crystals used in the experiment were of the LEAD type, which exhibit minimal temperature effects near room temperature. However, they are susceptible to humidity due to the adsorption of moisture on the electrode surface of the quartz crystal. The present experiments were conducted in a laboratory where the room temperature was maintained at around 20 °C, and the measurement time per experiment was 15–20 min, suggesting that significant fluctuations due to temperature changes were unlikely. However, in actual field measurements, temperature variations could still have an impact. Achieving accurate temperature control in field conditions may be challenging. Therefore, considering simplicity and power efficiency, it is preferable to use a configuration where the measuring section and other parts are placed within a heat insulator such as Styrofoam to minimize temperature effects.

In the current measurement setup (Figure 3), the device is designed to pass through a dehumidifier, maintaining a constant low-humidity environment. This configuration is also effective for the QCM-Hg sensor in reducing humidity effects. If humidity fluctuates significantly, the use of a dehumidifier (e.g., magnesium perchlorate), which is difficult to adsorb only mercury, may further enhance performance. When measuring actual sample water, the influence of gassing substances present in the sample, such as sulfide and ammonia compounds, must be considered. In the measurement configuration used in this study, there is a section for removing these gases, which contains a NaOH solution (Figure 3, Acid gas trap) that can neutralize substances that may interfere with mercury detection.

The present detection principle is based on the amalgamation reaction between elemental mercury (atomic mercury) and gold. This reaction occurs when mercury is vaporized and subsequently adsorbed onto the gold surface. Therefore, unless other heavy metal ions are similarly vaporized and adsorbed onto the gold surface, they do not interfere with the detection. Additionally, mercury present in aqueous solutions is detected in its gaseous form through a reduction-vaporization method, and thus, at this stage, the influence of other heavy metal ions is negligible.

However, in some cases, mercury in solution may react with other heavy metal ions to form species that are not in the elemental mercury form. In practice, as exemplified by hot spring water, numerous naturally occurring substances that may interfere with mercury measurement can be present. To eliminate such interfering substances, it is necessary to apply appropriate pretreatment—specifically, chemical processing commonly used in environmental analysis—in order to reduce mercury to its elemental state [53,54,55].

A comparative overview of major mercury detection methods, including QCM-based sensing, AAS, and gas detection tubes, is summarized in Table 1, highlighting the advantages and limitations of each technique in terms of sensitivity, cost, and field applicability.

## 5. Future Perspectives and Challenges

We have evaluated the basic characteristics of a QCM-based mercury detection system. The detection method employed in this system relies on the amalgam reaction between the gold electrode of the QCM and mercury, converting the mass change on the electrode surface into a frequency shift.

The key test results are as follows:

1. A proportional relationship exists between mercury concentration and the change in oscillation frequency.
2. Higher measurement flow rates and longer measurement times promote the reaction, leading to increased frequency changes. However, the rate of frequency change does not increase significantly with further increases in flow rate (250 mL/min).
3. The detection system requires optimization using a measurement unit (flow cell).

These results demonstrate that a QCM-based mercury detection method, when combined with optimized measurement conditions, enables high-sensitivity detection.

Specifically, using a quartz crystal element with a fundamental frequency of 30 MHz, and under experimental conditions of a flow rate of 100 mL/min and a measurement duration of 10 min, an approximation equation (Equation (3)) was derived based on the frequency shifts corresponding to each mercury concentration. Applying this equation, a mercury concentration of 1 µg/m^3^ yields a frequency shift of approximately 5 Hz after 10 min of measurement. Furthermore, as shown in Figure 7, the experimental results indicate that the frequency shift increases with higher flow rates.

Based on this approximation equation and the experimental findings, it was demonstrated that the QCM-Hg sensor can detect mercury at concentrations as low as approximately 1 µg/m^3^—a level that has previously only been achievable with commercially available analytical instruments. Moreover, detection of mercury concentrations below 1 µg/m^3^ is also considered feasible.

This study mainly focused on the experimental results obtained within the concentration range up to 100 µg/m^3^. Within this range, a linear relationship between mercury concentration and frequency shift was maintained, suggesting the sensor’s suitability for quantitative analysis. The primary objective of developing the QCM-Hg sensor is to detect low concentrations of mercury in field environments, specifically in situations where conventional gas detection tubes are unable to provide reliable measurements. Notably, the lower detection limit of commonly used gas detection tubes is typically around 50 µg/m^3^.

Therefore, if linearity can be confirmed up to 100 µg/m^3^, the QCM-Hg sensor would be expected to serve as a practical and sensitive tool for on-site mercury monitoring.

The quantification of mercury in aqueous samples was demonstrated to be feasible through a measurement system integrating the reduction-vaporization method with a QCM-Hg sensor. In this experiment, a water sample containing 5 ng of mercury (0.5 µg/L), which corresponds to the Japanese environmental water quality standard, was used. Assuming that the entire amount of mercury was vaporized and introduced into a 1 L sampling bag, the resulting gaseous mercury concentration would be equivalent to 5 µg/m^3^. By applying approximation Equation (3), derived for gas-phase mercury measurements, this concentration corresponds to a frequency shift of approximately 25 Hz. These results indicate that the system is capable of detecting mercury concentrations at the level of the water quality standard. Furthermore, based on the previously demonstrated gas-phase detection sensitivity [23] and the present experimental findings, it is expected that increasing the volume of the water sample would lead to a higher amount of vaporized mercury, thereby enabling detection of mercury concentrations below the regulatory limit.

This level of detection sensitivity is comparable to that of existing analytical instrumentation methods (portable instruments based on AAS). The results suggest that the measurement of mercury in water samples, which could previously only be performed in analysis laboratories, can now be conducted on-site. Furthermore, current measurement instruments employing the reduction-vaporization method rely on high-cost components in the absorbance detection system—such as the light source and photodetector—resulting in a high overall system cost. Therefore, replacing the costly absorbance detection unit—comprising the light source and photodetector—with a QCM-Hg sensor could significantly reduce the overall system cost. Additionally, as the QCM-Hg sensor is capable of miniaturization, it is expected to be more feasible for use in developing countries, where access to analytical equipment has traditionally been limited.

Thus, the present method utilizing the QCM-Hg sensor offers several advantages over conventional measurement instruments. Specifically, it enables the detection of mercury at environmentally relevant concentrations using a compact and cost-effective quartz crystal element. The small size and lightweight design of the device make it highly portable and convenient for transport. Moreover, field measurements are feasible with battery-powered operation, and the system demonstrates excellent practicality owing to its simple and user-friendly interface compared to traditional analytical equipment.

In this study, a new quartz crystal element was used for each experiment. This decision was made because the experimental conditions—such as mercury concentration and measurement duration—varied across experiments, potentially leading to differences in the surface condition of the electrodes after each measurement. However, from a cost perspective, using a new element for every measurement increases the total number of elements required, which may result in higher overall costs.

The QCM-Hg sensor developed in this study is intended for the on-site measurement of mercury concentrations at or below environmental regulatory levels. Measurement instruments currently available for such applications are often more than 1000 times more expensive than the quartz crystal element used in our system. In practical field applications, gas detection tubes are commonly used, and the disposable nature of their sensing elements is generally accepted. Although the unit cost of a quartz crystal element is several times higher than that of a gas detection tube, the latter cannot detect mercury concentrations at environmentally relevant levels.

Therefore, considering both performance and cost, the QCM-Hg sensor offers a practical and cost-efficient solution for field deployment.

Thus, the QCM-Hg sensor developed in this study constitutes a compact and practical system capable of rapidly measuring mercury concentrations at regulatory levels, which has traditionally been difficult under field conditions. In the context of assessing mercury pollution, in addition to short-term measurements at regulatory thresholds, long-term monitoring—such as personal exposure assessments over approximately 8 h—is also a critical issue. Although the QCM-Hg sensor is, in principle, applicable to long-term measurements, several technical challenges remain for its practical implementation. In our research and development, we prioritized short-term measurements (typically within one hour) in light of these considerations. One of the main challenges associated with long-term measurements is the influence of environmental factors such as temperature and humidity. While these factors also affect short-term measurements, their relative impact is generally smaller due to the shorter duration. In contrast, during prolonged measurements, the cumulative effects of environmental fluctuations become more significant, necessitating appropriate correction mechanisms. Although such correction strategies are technically feasible at present, their implementation could lead to system enlargement and increased cost, potentially compromising the inherent advantages of the QCM-Hg sensor, such as portability and simplicity. Moreover, interferences from coexisting gases or airborne particulates—which are negligible in short-term measurements—may become more pronounced in extended monitoring. Mitigation of these additional influences may also introduce drawbacks, including increased system complexity and cost. It should be noted that such environmental interferences are not unique to the QCM-Hg sensor, but are common challenges among environmental sensing technologies in general. It is therefore anticipated that future technological advancements will help overcome these limitations and enable practical long-term monitoring with such sensors.

This review has summarized both the theoretical underpinnings and practical demonstrations of the QCM-Hg detection system developed by the authors. By contextualizing the presented findings within the broader landscape of mercury monitoring technologies, this article aims to bridge fundamental principles with application-level insights, particularly for low-cost, field-deployable environmental sensing.

The implementation of this system could contribute to the goals of the Minamata Convention on Mercury [4], which Japan is actively promoting, by facilitating on-site mercury monitoring and reducing the reliance on costly analytical systems.

## Figures and Tables

**Table 1:** Comparison of representative mercury detection methods in terms of sensitivity, cost, portability, and applicability for field use.

Detection Method	Principle	Detection Limit	Ref.
QCM-Hg	Mass change via amalgamation on Au electrode	~1 µg/m^3^ (gas), ~0.05 µg/L (liquid)	[21,23]
AAS	Atomic absorption spectroscopy	~0.1 µg/m^3^	[11,12,13,14]
Gas detection tube	Colorimetric chemical reaction	>50 µg/m^3^ (gas), >5 µg/L (liquid)	[15,16]
CVAFS	Cold vapor atomic fluorescence spectroscopy	~0.01 µg/L	[19]
Electrochemical	Electrochemical detection using modified electrodes and voltammetry	~0.1 nM	[56,57,58]
Aptamer-based sensor	Thymine binding induces aptamer structural change	~3 nM	[59,60]

## Data Availability

This review article is based primarily on previously published studies. All data shown in the figures are derived from those publications, except for the schematic illustrations (e.g., Figure 1 and Figure 4), which were newly created by the authors. For further information, readers may contact the corresponding author.
